# Bioinformatic analysis and validation of microRNA‐508‐3p as a protective predictor by targeting NR4A3/MEK axis in pulmonary arterial hypertension

**DOI:** 10.1111/jcmm.16523

**Published:** 2021-05-04

**Authors:** Yi Ma, Shu‐Shu Chen, Fen Jiang, Ru‐Yi Ma, Huan‐Liang Wang

**Affiliations:** ^1^ Department of Anesthesiology Qilu Hospital of Shandong University Jinan China; ^2^ The Key Laboratory of Cardiovascular Remodeling and Function Research Chinese Ministry of Education Chinese National Health Commission and Chinese Academy of Medical Sciences The State and Shandong Province Joint Key Laboratory of Translational Cardiovascular Medicine Qilu Hospital of Shandong University Jinan China; ^3^ Shenzhen Research Institute of Shandong University Shenzhen China

**Keywords:** diagnosis biomarker NR4A3, MiR‐508‐3p, pulmonary arterial hypertension

## Abstract

Pulmonary arterial hypertension (PAH) featured a debilitating progressive disorder. Here, we intend to determine diagnosis‐valuable biomarkers for PAH and decode the fundamental mechanisms of the biological function of these markers. Two mRNA microarray profiles (GSE70456 and GSE117261) and two microRNA microarray profiles (GSE55427 and GSE67597) were mined from the Gene Expression Omnibus platform. Then, we identified the differentially expressed genes (DEGs) and differentially expressed miRNAs (DEMs), respectively. Besides, we investigated online miRNA prediction tools to screen the target gene of DEMs. In this study, 185 DEGs and three common DEMs were screened as well as 1266 target genes of the three DEMs were identified. Next, 16 overlapping dysregulated genes from 185 DEGs and 1266 target gene were obtained. Meanwhile, we constructed the miRNA gene regulatory network and determined miRNA‐508‐3p‐NR4A3 pair for deeper exploring. Experiment methods verified the functional expression of miR‐508‐3p in PAH and its signalling cascade. We observed that ectopic miR‐508‐3p expression promotes proliferation and migration of pulmonary artery smooth muscle cell (PASMC). Bioinformatic, dual‐luciferase assay showed NR4A3 represents directly targeted gene of miR‐508‐3p. Mechanistically, we demonstrated that down‐regulation of miR‐508‐3p advances PASMC proliferation and migration via inducing NR4A3 to activate MAPK/ERK kinase signalling pathway. Altogether, our research provides a promising diagnosis of predictor and therapeutic avenues for patients in PAH.

## 1 INTRODUCTION

Pulmonary arterial hypertension (PAH) is a cure‐resist and chronic disease with enigmatic molecular mechanisms.^1^ The aetiopathogenesis of PAH chiefly characterized by proliferation, migration, anti‐apoptosis or phenotype switches of pulmonary artery smooth muscle cell (PASMC), and leading to an abnormal elevation in pulmonary arterial pressure.^2^, ^3^, ^4^ Current treatment strategies for this disorder are palliative and do not significantly improve long‐term survival.^5^, ^6^, ^7^ Therefore, new therapies’ plan is urgent to be developed, which deliver more tangible benefits to patients in PAH.

In recent years, microarray has been reported effective in detecting the complex network during the process of PAH, and in screening biomarkers for PAH diagnosis and prognosis. Gene Expression Omnibus (GEO) is an online database containing millions of gene profiles in various diseases, and the GEO datasets could be used to determine differentially expressed genes (DEGs) and to establish miRNA‐mRNA regulatory networks.^8^ In this article, the mRNA microarray datasets were processed for further exploring. Dysregulated genes in PAH samples were examined to screen vital predictors. They were investigated via bioinformatic tools: Gene Ontology (GO) annotation, Kyoto Encyclopedia of Genes and Genomes (KEGG) pathway enrichment, protein–protein interaction (PPI) network and gene set enrichment analysis (GSEA). Finally, miR‐508‐3p and its responsive gene NR4A3 represent the highest interaction relation, implying miR‐508‐3p‐NR4A3 acts as an essential regulator in the progression of PAH.

miRNA, a type of non‐coding RNA, is a length of approximately 22 nucleotides, which negatively regulates the expression of post‐transcriptional genes by inhibiting the translation of target mRNA or by partially binding to the mRNA’s 3′ untranslated region (UTR) to degrade the mRNA.^9^, ^10^ Currently, miRNAs are widely recognized to associate with biological progression in PASMCs and endothelial cells. Studies also report that the abnormally expressed miRNAs are engaged in the pathogenesis of PAH by functioning as a pro‐PAH or anti‐PAH factor.^11^, ^12^, ^13^, ^14^, ^15^ However, the detailed mechanism of miRNA in PAH remains largely unformulated. To this end, we examined the miRNA gene inter‐regulation network by using bioinformatic methods and found that miR‐508‐3p was down‐regulated in PAH tissues. The role of miR‐508‐3p in PAH, however, remains relatively unknown.

It is exciting and vital that the functional expression of miR‐508‐3p in PAH and whether miR‐508‐3p could bind and regulate NR4A3 and what are the biological consequence of miR‐508‐3p‐NR4A3 axis. For now, there are more research needed to answer these questions. In this study, we demonstrate that miR‐508‐3p is significantly down‐regulated in PAH. Furthermore, miR‐508‐3p acts as a potential anti‐PAH‐miR that inhibits PASMC proliferation and migration by targeting NR4A3.

## 2 MATERIALS AND METHODS

### 2.1 Preparation and procession of raw datasets

**FIGURE 1:**
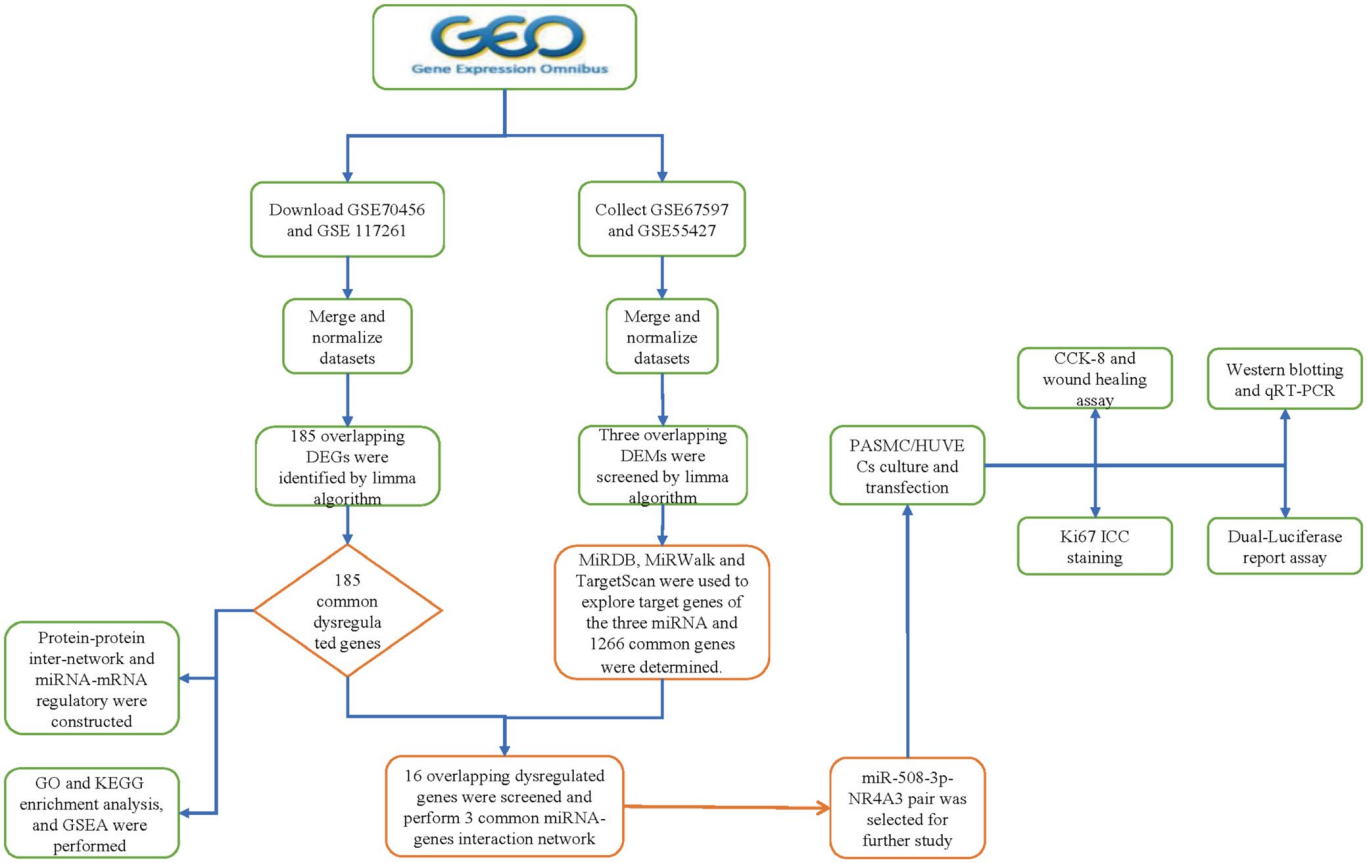
The detailed workflow of our study procedure

Series matrix files of GSE70456, GSE117261, GSE55427 and GSE67597 were downloaded from the GEO (http://www.ncbi.nlm.nih.gov/geo) website.^16^ Two mRNA expression profiling include 99 PAH patients and healthy controls. The microRNA (miRNA) microarray data consist of 27 PAH patients and healthy controls. All the samples we screened from the microarray are human‐original sequenced tissue profiling (the detailed information about four microarray profiling see Table 1). Then, the combined data were possessed by SVA package in R (4.0.2) to correct batch effects.^17^ A limma‐based rule was operated to screen DEGs from two mRNA microarrays and differentially expressed miRNAs (DEMs) from two miRNA datasets.^18^, ^19^ We regarded an adjusted *P* < .05 and |log FC| > 1.5 as statistically significance. To make this paper more clearly, we illustrated data processed procedure in the workflow plot (Figure 1).

**TABLE 1:** The information on microarray expression profiling

Microarray	Type	Platform	Sample(s)
GSE70456	mRNA	GPL15207	16
GSE117261	mRNA	GPL6244	83
GSE55427	miRNA	GPL18346	12
GSE67597	miRNA	GPL18402	15

### 2.2 GO annotation and KEGG pathway enrichment analysis

Gene Ontology (GO) analysis was applied to deeper exploring the potential biological process (BP), molecular function (MF) and cellular component (CC) of DEGs.^20^ The Kyoto Encyclopedia of Genes and Genomes (KEGG) pathway enrichment analysis of the DEGs was submitted to R to conduct functional and signalling pathway analysis.^21^, ^22^ Gene count >2 and *P* < .05 were considered to be statistically significant.

### 2.3 Protein‐protein interaction (PPI) network and Hub module identification

To systematically analyse the biological functions of DEGs, online tool STRING database (https://string‐db.org/) was employed with a combined score >0.9 was set as the cut‐off point. Then, the Cytoscape (3.8.1) was applied for constructing and visualizing the complicate regulatory network of DEGs.^23^ Significant modules and hub genes in the PPI network were pinpointed by MCODE (1.6.1) and cytoHubba, a plug‐in of Cytoscape. The parameters of DEGs clustering and scoring were set as follows: MCODE score ≥ 4, degree cut‐off = 2, node score cut‐off = 0.2, max depth = 100 and *k*‐score = 2.

### 2.4 Construction of the mRNA‐miRNA interaction network

The common DEMs were acquired from two miRNA datasets, respectively. To construct and analyse the predominant miRNA‐mRNA regulatory network, we investigated the online RNA‐prediction tools: TargetScan (http://www.targetscan.org/vert_72/), miRDB (http://mirdb.org/) and miWalk (http://mirwalk.umm.uniheidelberg.de/) to predict the target mRNAs of common DEMs.^9^ Then, we combined DEGs with miRNA target genes and obtained the overlapping genes. Finally, we used Cytoscape to construct and visualize the interaction network of mRNAs and related miRNAs.

### 2.5 Gene set enrichment analysis (GSEA)

GSEA (4.1.0) was used to analyse the data. Enrichment analysis was performed between the PAH and control groups using default weighted enrichment statistics.^24^ c5.go.v7.2.symbols.gmt [Gene ontology] and c2.cp.kegg.v7.2.symbols.gmt [curated gene sets] were set as the explored enriched gene database with gene set permutations as 1000 times in each group.

### 2.6 Cell culture and transfection

Human pulmonary arterial smooth muscle cell (H‐PASMC; ATCC^®^ PCS‐100‐023™) and human umbilical vein endothelial cells (HUVECs; ATCC^®^ PCS‐100‐010™) were purchased from American Type Culture Collection (ATCC). H‐PASMC and HUVECs were cultured in Dulbecco's modified Eagle medium (4.5 g/L D‐glucose; DMEM, ScienCell), both supplemented with 10% foetal bovine serum (FBS, Gibco) and 100 units/mL penicillin/streptomycin in a humidified incubator at 37°C with 5% CO_2_. The miR‐508‐3p mimics, miR‐508‐3p inhibitor, normal control (NC) oligonucleotides and the small‐interfering RNA (siRNA) targeting NR4A3 mRNA kit were purchased from GenePharma. Cell transfection was performed with Lipofectamine™ 2000 transfection reagent (Invitrogen) according to the manufacturer's instructions.

### 2.7 Cell proliferation assay

Cell proliferation assays were measured using a cell counting kit‐8 (CCK‐8, Dojindo Laboratory) according to the manufacturer's instruction. Briefly, cells were seeded in 96‐well plates at 5.0 × 10^3^ cells/well and cultured for 2 days. We set the checkpoint at 1, 2, 4, 8, 12, 24 and 48 hours, respectively. At the indicated times, 10 μL of CCK‐8 solution was added to each well and incubated for an additional one h at 37°C. The absorbance of the mixed solutions was measured at 450 nm with a SpectraMax M5 Multi‐Mode Microplate Reader (Molecular Devices LLC).

### 2.8 Wound healing assays

For migration assays, cells were grown in a six‐well plate. As the cells reached 70%‐80% confluence, a scratch was performed with a micropipette tip followed by wash three times, used sterile phosphate‐buffered solution (PBS), and cultured with 10% FBS with Nocodazole (HY‐13520, MCE, USA) in DMEM. Then, the scratch width was observed at 0 and 48 hours and photographed by a microscope. Image J was used to analyse scratch closure per cent.

### 2.9 Immunocytofluorescense assay (ICC)

PASMC was cultured 24 hours after treated with miR‐508‐3p mimics, inhibitor or NC. Then, the cells were fixed with 4% paraformaldehyde for 15 minutes and washed three times with PBS, followed by permeabilized with 0.1% Triton X‐100 for 20 minutes. After washing three times with PBS, cells were blocked with 3% FBS for one h at room temperature. They were incubated with primary antibody against Ki67, a marker of cell proliferation, (ab16667, 1:100, Abcam) at 4°C overnight, respectively. Then, the Alexa Fluor 594‐conjugated second antibody was applied at 37°C for 1 hour. After three washes with PBS, nuclei were stained with DAPI (AR1176, BOSTER) for 15 minutes at room temperature. Finally, cell images were examined in a darkroom using an electron microscope (Nikon).

### 2.10 Quantitative real‐time PCR (qRT‐PCR)

Total RNA was extracted from PASMC/HUVEC or PAH rat pulmonary artery tissue using TRIzol (Invitrogen) according to the manufacturer's instruction. For the mRNA expression test, 1 μg of total RNA was used to synthesize cDNA using a PrimeScript™ RT reagent kit (No. RR037A, TaKaRa) and the cDNA was used for quantitative PCR with the TB Green Premix Ex Taq™ kit (No. RR420, TaKaRa). For miRNA expression test, 1μg of total RNA extracted from HPASMC/HUVEC and miR‐508‐3p primer or U6 primer was employed to reverse transcribe with PrimeScript™ RT reagent. Then, the RT products were applied to quantitative PCR with TB Green Premix Ex Taq™ kit (No. RR420, TaKaRa). We set the thermal cycler conditions as follows: 30 seconds at 95.0℃ for cDNA denatured, 40 cycles of 95.0℃ for 5 seconds, 60℃ for 30 seconds and 1 minutes at 60.0℃. Each sample was performed in triplicate. GAPDH and U6 were referred to as internal control to calculate the relative gene expression and to calculate the relative miR‐508‐3p term, respectively. Both miRNA and gene expression level measured by qRT‐PCR were presented as 2^−ΔΔCT^. The primers for miR‐508‐3p, U6 and other detective genes are listed in Table 2.

**TABLE 2:** The primer sequences for qRT‐PCR designed in this study

Gene symbol	Sequence (5′ to 3′)
miR‐508‐3p‐F	ACTGTATGATTGTAGCCTTTTGG
miR‐508‐3p‐R	TATGGTTTTGACGACTGTGTGAT
miR‐508‐3p mimics	UGAUUGUAGCCUUUUGGAGUAGA UACUCCAAAAGGCUACAAUCAUU
miR‐508‐3p inhibitor	UCUACUCCAAAAGGCUACAAUCA
miR‐inhibitor NC	CAGUACUUUUGUGUAGUACAA
miR‐298‐F	TCAGGTCTTCAGCAGAAGC
miR‐298‐R	TAGTTCCTCACAGTCAAGGA
miR‐632‐F	GACGGGAGGCGGAGCGGGGA
miR‐632‐R	TCCCCGCTCCGCCTCCCGTC
U6‐F	CAGCACATATACTAAAATTGGAACG
U6‐R	ACGAATTTGCGTGTCATCC
NR4A3‐F	GCGTCCAAGCCCAATATAGC
NR4A3‐R	CGTGGTGTATTCCGAGCTGT
siNR4A3#1	CCUUCCUGCGUGUACCAAATT UUUGGUACACGCAGGAAGGTT
siNR4A3#2	GCCCUUGUCCGAGCUUUAATT UUAAAGCUCGGACAAGGGCTT
siNR4A3#3	CCCUGGUAGAACUGAGGAATT UUCCUCAGUUCUACCAGGGTT
si‐negative control	UUCUCCGAACGUGUCACGUTT ACGUGACACGUUCGGAGAATT
GAPDH‐F	GCACCGTCAAGGCTGAGAAC
GAPDH‐R	TGGTGAAGACGCCAGTGGA

Note

GAPDH was viewed as an internal control to calculate the relative gene expression. U6 was used as a reference control to calculate the relative miR‐508‐3p term; F, forward; R, reverse.

### 2.11 Western blot analysis

The total protein was harvested and lysed in RIPA buffer and was measured using a Bicinchoninic Acid Assay kit (BOSTER). The protein samples were separated by 10% sodium dodecyl sulphate‐polyacrylamide gel electrophoresis followed by transfer to polyvinylidene difluoride membranes. After blocked in 5% non‐fat milk for 2 hours at room temperature, the membranes were incubated with primary antibodies overnight at 4°C. The primary antibodies target ERK (1:1000), p‐ERK (1:1000), Slug (1:1000) and GAPDH (1:3000) were purchased from Cell Signal Technology. NR4A3 (1:500; sc‐393902) was purchased from Santa Cruz Biotechnology. Then, the membranes were incubated with the responsive HRP‐coupled secondary antibody (1:5000) for 2 hours at room temperature, and protein bands were analysed by Image J.

### 2.12 Dual‐luciferase reporter assay

The putative miR‐508‐3p wild‐type and mutant sequences in the 3′ UTR of NR4A3 gene were cloned into a pmirGLO‐firefly luciferase reporter vector (Genechem) for NR4A3. The established report vectors coupled with renilla vector, and miR‐508‐3p mimics or miR‐NC were cotransfected into HPASMC/HUVEC. After cultured for 24 hours, the transfected cells were measured the luminescence signals with a Luminometer (TD‐20/20) according to the instruction of Dual‐luciferase Reporter Assay System kit (Promega). The renilla luciferase signal intense was regarded as normalization.

### 2.13 Animal model

Male Sprague‐Dawley rats weighing (180‐200 g) were purchased from Shandong University and divided into two groups (n = 5 for each group) as follows: the control group and monocrotaline (MCT) group. MCT‐induced PAH model was developed by a single subcutaneous injection of MCT (60 mg/kg; C2401, Sigma‐Aldrich) in a consecutive 2 weeks.^25^ Control group rats received saline. Our research was conducted abided by the principles of the Laboratory Animal Ethics Committee of Shandong University.

### 2.14 Statistical analysis

Data are presented as mean ± standard. Student's *t* test was used to analyse the differences between two groups, and more than two groups were performed with one‐way ANOVA followed by a Tukey‐Kramer post hoc test. All of the statistical analyses and plots were performed by GraphPad Prism 8.0. software. Image J was used to analyse cell migration and protein expression level. *P*‐value < .05 was considered a statistically significant.

## 3 RESULTS

### 3.1 DEGs and DEMs identified from the raw datasets

**FIGURE 2:**
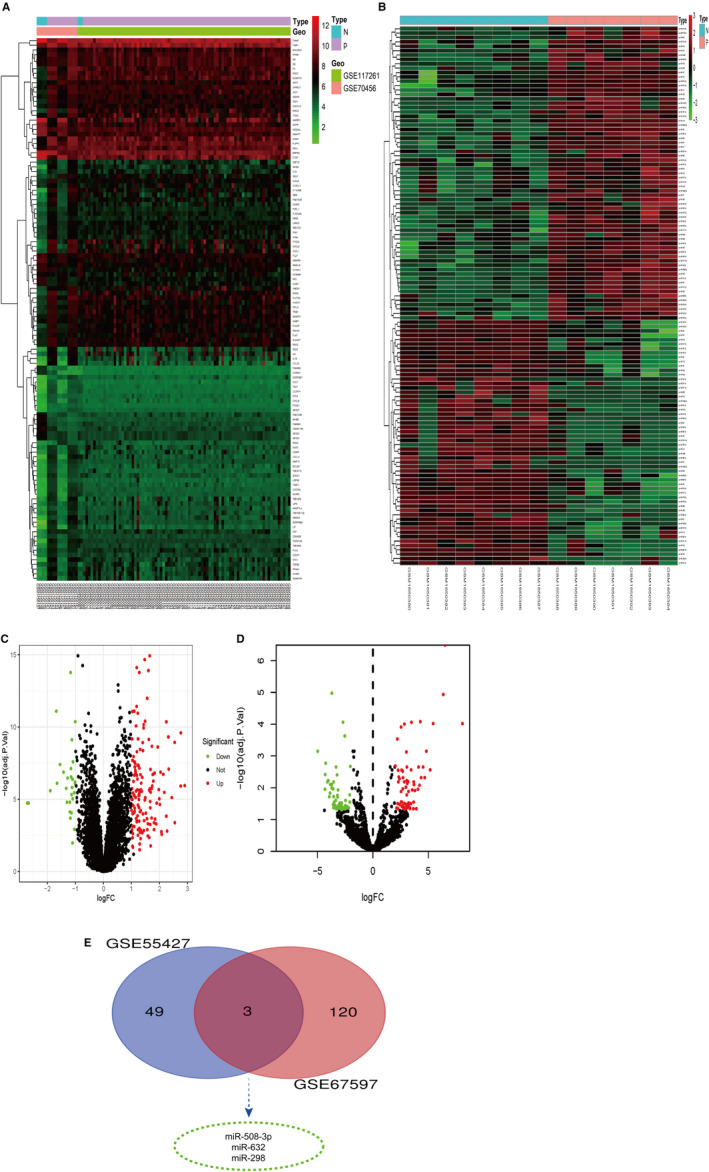
The DEGs and DEMs of four microarrays profiles. A, The heatmap of DEGs. B, The heatmap of DEMs. C, Dysregulated genes in mRNA datasets presented in volcano plot with ‘green’ symbolized low expression, ‘red’ demonstrated high expression. D, The miRNA in miRNA datasets presented in volcano plot. E, Venn diagram to show the common miRNA of DEMs from two miRNA microarrays. DEGs, differentially expressed genes; DEMs, differentially expressed miRNAs

**FIGURE 3:**
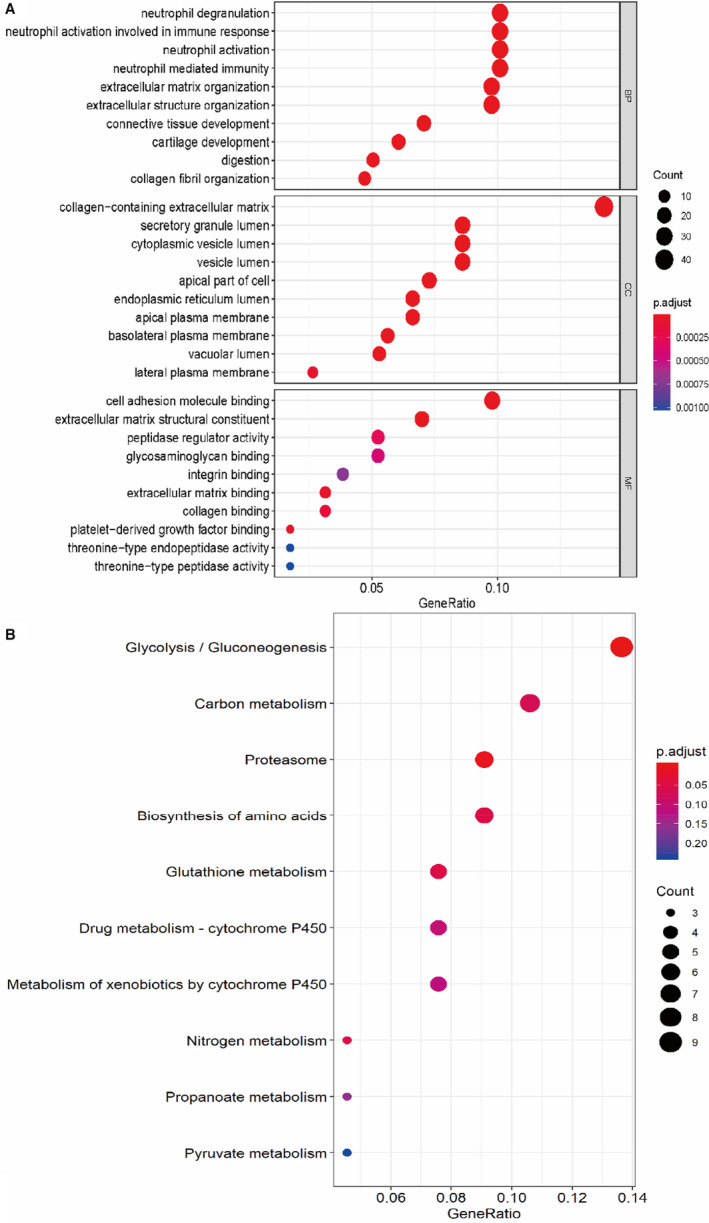
The results of GO annotation and KEGG pathway analysis. A, The outcomes of GO, which consists of three parts: biological process, cellular component and molecule function. B, The main signalling pathway identified using KEGG. GO, Gene Ontology; KEGG, The Kyoto Encyclopedia of Genes and Genomes

**FIGURE 4:**
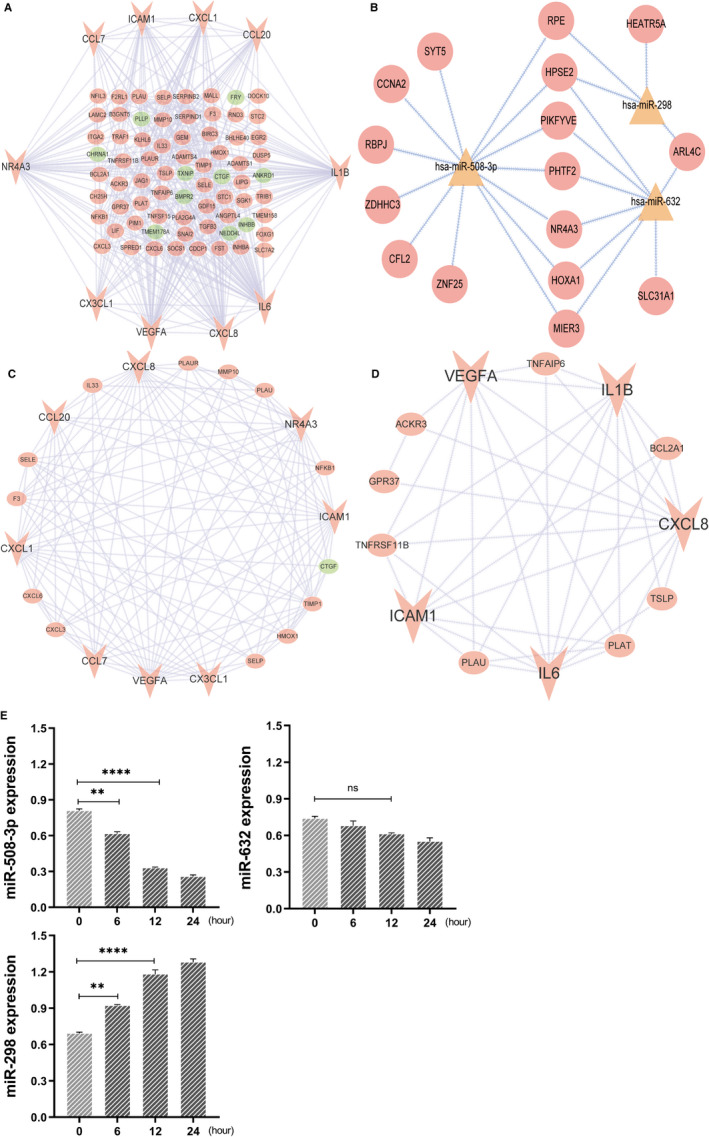
Protein‐protein interaction and mRNA‐miRNA regulatory network construction. A, The interconnection of protein‐protein and ‘green’ stand for down‐expression genes while ‘red’ represents up‐expression genes, ‘triangle’ shape symbolized the top ten genes. B, MicroRNA‐mRNA interacts network. ‘triangle’ shape represented microRNA, and ‘circle’ shape indicated target genes. C, The first module identified in PPI, which ‘green’ indicated down‐expression genes while ‘red’ represents up‐expression genes, and ‘triangle’ shape symbolized the top ten genes. D, The second module screened in PPI, which ‘triangle’ shape suggested the top ten genes. E, The expression of three miRNAs in PASMC stimulated by PDGF at different time‐points (n = 3, normalized to U6, **P < .01, ***P < .001, compared to 0 h. ns, no difference). PPI, protein–protein interaction

**FIGURE 5:**
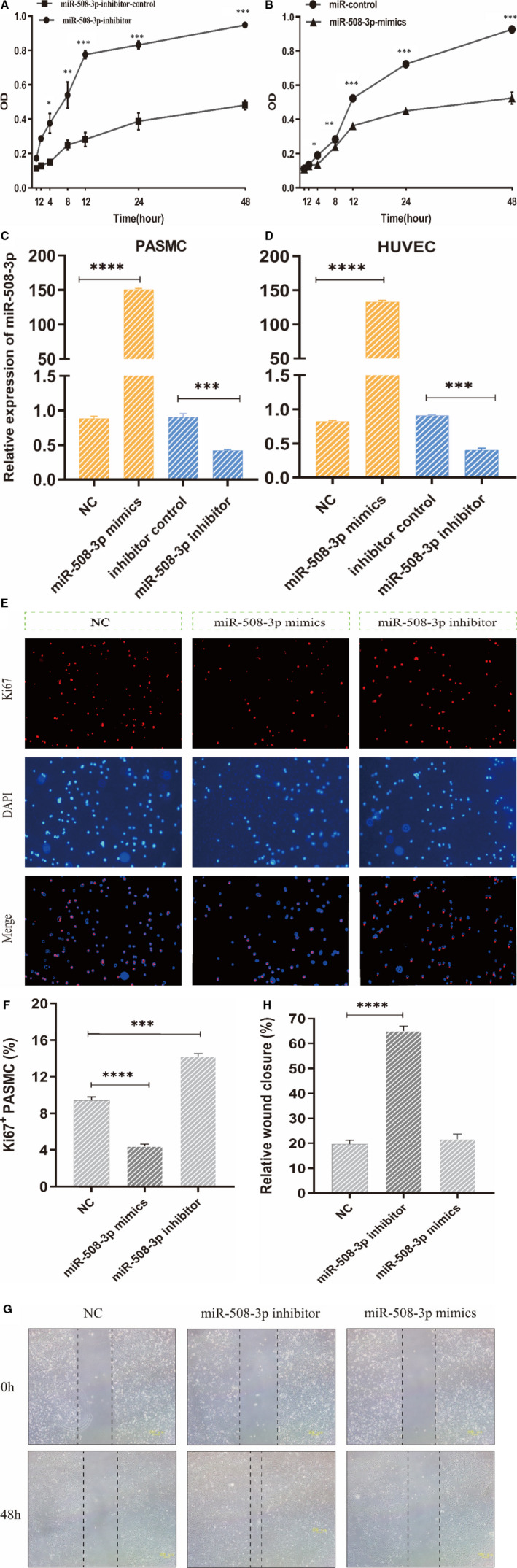
miR‐508‐3p enhances proliferation and migration of PASMC. A‐B, In CCK‐8 assay, PASMC with down‐expressing miR‐508‐3p represents high proliferation rate while PASMC transfected miR‐508‐3p mimics showed a significantly lower cell proliferation rate compared with the control cell (n = 3). C‐D, qRT‐PCR indicated that miR‐508‐3p represents higher or lower expression in PASMC and HUVEC transfected with miR‐508‐3p mimics or inhibitor compared with the control cells (n = 3). E‐F, In immunocytofluorescence assay, PASMC treated with miR‐508‐3p NC or miR‐508‐3p mimics/inhibitor and cultured for 24 h, then, Ki67, a marker of cell proliferation, as shown in red and DAPI was in blue. Image‐Pro Plus 6.0 was operated to measure the number of DAPI and Ki67^+^ nuclei. The ratio of Ki67 staining was defined as the percentage of positive nuclei within the total (DAPI staining plus Ki67^+^ staining) number of nuclei (n = 3). G‐H, In scratch wound healing assay, PASMC with transfection of miR‐508‐3p inhibitor or mimics showed a significantly accelerated wound healing in down‐expressed miR‐508‐3p cells compared with the control cells (n = 3). *P < .05, **P < .01 and ***P < .001

**FIGURE 6:**
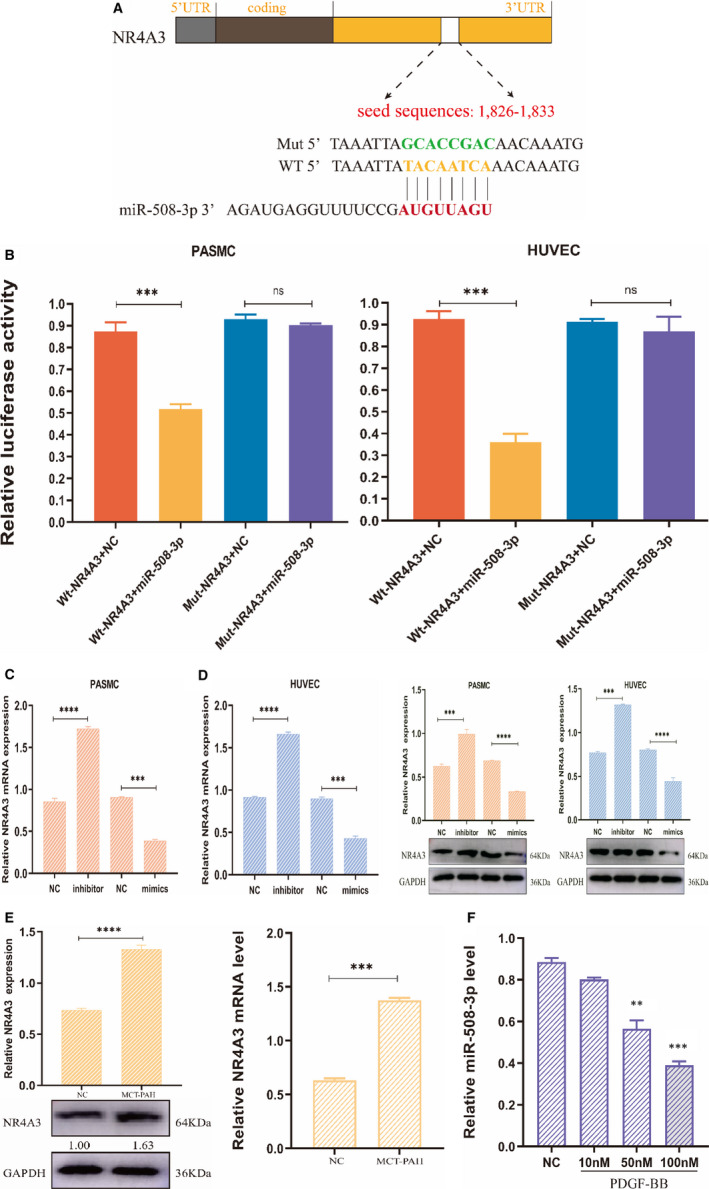
miR‐508‐3p targets NR4A3 in PASMC/HUVEC. A, The interaction model of miR‐508‐3p and NR4A3. The binding seed sequence (in red) of miR‐508‐3p in 3′UTR of NR4A3 mRNA as predicted by the TargetScan online tool, and the mutated seed sequence (in green) in the same 3′UTR, and the seed sequence in miR‐508‐3p (in red). B, In dual‐luciferase report assay, PASMC/HUVEC were transfected with a plasmid vector, which containing wild‐type 3′UTR of NR4A3 and miR‐508‐3p mimics, showed a significant reduction in luciferase intensity compared with the control cells. In contrast, the cells were transfected with a plasmid vector coupled with the mutated 3′UTR of NR4A3 and miR‐508‐3p mimics. The luciferase signal intensity showed no reduction compared with the control cells (n = 3). From qRT‐PCR and protein expression level, after PASMC (C) and HUVEC (D) transfected with miR‐508‐3p mimics, the expression levels of NR4A3 shown significantly low; on the contrary, the cells were transfected with miR‐508‐3p inhibitor, and the expression level of NR4A3 was enhanced compared with the control cells (n = 3, normalized to GAPDH). E, In MCT‐PAH rat pulmonary artery tissues, NR4A3 was higher expressed at mRNA and protein level compared with the NC group (n = 5, each group, normalized to GAPDH). F, In the qRT‐PCR assay, miR‐508‐3p was a lower expression in PASMC treated with a various dose of PDGF‐BB compared with the control cells (n = 3, normalized to U6). The values labelled under protein bands represent the relative intensity of the bands. (*P < .05, **P < .01 and ***P < .001)

**FIGURE 7:**
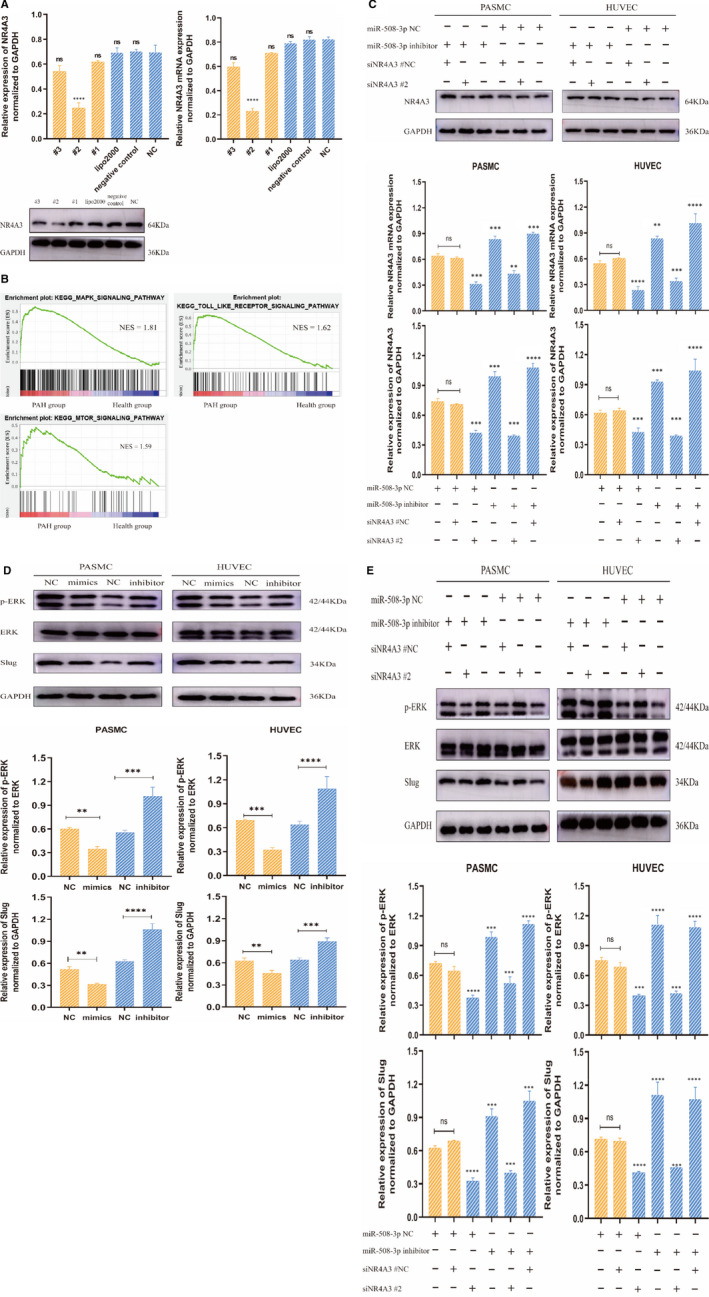
miR‐508‐3p down‐expression contributes aggressive PASMC proliferate in PAH via activation of NR4A3/MEK pathway. A, To knock down NR4A3 expression, we tested the efficiency of candidate siRNA target NR4A3 at the mRNA and protein level and selected siRNA#2 as the most effective siRNA for further study (n = 3, compared to control). Lipo2000 means lipo‐transfection reagent 2000. B, GSEA analysis showed that PAH groups were primarily involved in activating MAPK, Toll‐like receptor and mTOR signalling pathways. NES: normalized enrichment score. C, PASMC and HUVEC treated with miR‐NC and siRNA# 2 against NR4A3 compared with that transfected with miR‐NC and siRNA‐NC, the expression of NR4A3 was decreased at mRNA and protein level; as these cells were transfected with miR‐508‐3p inhibitor and siRNA‐NC, NR4A3 expression was increased; when these cells were sequentially knockdown miR‐508‐3p by inhibitor and NR4A3 by siNR4A3#2, NR4A3 expression was down‐regulated again (n = 3, compared to the first two groups). D, Western blotting analysis indicated that transfection of miR‐508‐3p mimics in PASMC and HUVEC decreased the expression level of p‐ERK and Slug. However, these cells transfected with miR‐508‐3p inhibitors showed the reverse results compared with the control cell (n = 3, compared to NC). E, The Western blotting revealed that the protein levels were decreased when PASMC and HUVEC were knockdown of NR4A3; as merely miR‐508‐3p was down‐regulated by miR‐508‐3p inhibitor, the protein levels of p‐ERK and Slug were increased; when these cells were treated by knockdown of miR‐508‐3p and NR4A3, the p‐ERK and Slug were decreased (rescued) again (n = 3, compared to the first two groups). (ns: no difference, *P <.05, **P <.01 and ***P <.001)

The four microarrays profiling were subjected to merge and normalize; then, DEGs and DEMs were screened using limma algorithm. In total, 185 dysregulated expression gens were obtained based on the combined datasets, including 163 up‐regulated and 22 down‐regulated DEGs. Besides, 172 DEMs were acquired with 81 down‐regulated and 91 up‐regulated DEMs. Next, different mRNA and miRNA expression between PAH cases and healthy controls were visualized by heatmap and volcano plots (Figures 2, 3, 4, 5, 6, 7). The common intersect of DEM between the two miRNA expression profiles was shown using a Venn diagram (Figure 2E).

### 3.2 GO enrichment and KEGG pathway analysis of DEGs

To determine biological features of DEGs, GO annotation and KEGG pathway analysis were accomplished by R as follows (Table 3): cellular component (CC) enrichment analysis showed the groups of genes were mainly enriched in the collagen‐containing extracellular matrix, biological process (BP) enrichment analysis showed that the DEGs primarily enriched appeared on neutrophil degranulation and neutrophil activation, and molecular function (MF) enrichment analysis indicated that the DEGs were enriched in cell adhesion molecule binding (Figure 3A). KEGG pathway enrichment analysis suggested that this group of genes was enriched in glycolysis and gluconeogenesis (Figure 3B).

**TABLE 3:** GO annotation and KEGG enrichment analysis of DEGs with degree >10

Term	Description	*p*‐adjust	Count
Biological process			
GO:0042119	Neutrophil activation	2.52E−07	30
GO:0002446	Neutrophil mediated immunity	2.52E−07	29
GO:0030198	Extracellular matrix organization	1.88E−09	29
GO:0043062	Extracellular structure organization	1.88E−09	29
GO:0002683	Negative regulation of immune system process	2.78E−06	26
Cellular component			
GO:0062023	Collagen‐containing extracellular matrix	3.38E−21	43
GO:0034774	Secretory granule lumen	7.38E−10	26
GO:0060205	Cytoplasmic vesicle lumen	7.38E−10	26
GO:0005788	Endoplasmic reticulum lumen	3.81E−06	20
GO:0016323	Basolateral plasma membrane	2.83E−06	17
Molecular function			
GO:0050839	Cell adhesion molecule binding	3.31E−06	28
GO:0005201	Extracellular matrix structural constituent	1.20E−09	20
GO:0048018	Receptor ligand activity	5.91E−05	19
GO:0030546	Signalling receptor activator activity	0.00882152	19
GO:0045296	Cadherin binding	0.00134589	17
KEGG			
hsa00010	Glycolysis/Gluconeogenesis	0.00026365	9
hsa01200	Carbon metabolism	0.00023003	8
hsa03050	Proteasome	0.00073212	7
hsa04010	MAPK signalling pathway	0.00052966	6

Abbreviations: DEGs, differentially expressed genes; GO, Gene Ontology; KEGG, The Kyoto Encyclopedia of Genes and Genomes; MAPK, mitogen‐activated protein kinase.

### 3.3 Protein‐protein inter‐regulatory network and miRNA‐mRNA network construction

To unveil the underlying interaction relations among DEGs, the STRING online tool was applied and visualized via Cytoscape (Figure 4A). We have identified ten critical gene signatures in the network, named IL6, CCL7, CCL20, IL1B, ICAM1, CX3CL1, CXCL1, CXCL8, NR4A3 and VEGFA. Also, we determined two crucial modules in the PPI network using MCODE, which contained 34 genes (Figure 4C‐D). Next, online bioinformatic platforms miRWalk, miRDB and TargetScan were used to predict target genes of the three commons miRNA, and a total of 1266 overlapping molecule was screened. Then, we have combined 185 DGEs and 1266 miRNA target genes to identify overlapping genes. Finally, 16 overlapping dysregulated genes were presented, and the internetwork linked these genes, and three common miRNAs were portrayed via Cytoscape (Figure 4B). The outcome indicated that miR‐508‐3p plays a crucial role in the regulatory network, and NR4A3 was identified as the core gene in the PPI network and the miRNA‐mRNA inter‐relation network. Indeed, several studies reported that NR4A3 serves as a significant player involved in vascular‐related disorders for it has been demonstrated that exert promote proliferation and migration in vascular smooth muscle cells, for instance, Atherosclerosis, Coronary artery disease. To identify the miRNAs’ necessary in regulating human h‐PASMC proliferation, we conducted miRNA‐related expression experiment in h‐PASMC at 0, 6, 12 and 24 hours after PDGF‐BB (50 nmol L^−1^ mL^−1^) stimulation (Table 4). Finally, hsa‐miR‐298 was up‐regulated, and hsa‐miR‐508‐3p and hsa‐miR‐632 were down‐regulated in response to PDGF stimulation, which is in agreement with bioinformatic analysis (Figure 4E). Besides, miR‐508‐3p was found to be highly enriched in h‐PASMC as well as its expression was markedly down‐regulated, in a time‐dependent manner, in response to PDGF‐BB treatment. Therefore, these findings indicate that miR‐508‐3p may be a dominant regulator for h‐PASMC proliferation. In consequence, the bio‐function and complicating mechanisms of miR‐508‐3p‐NR4A3 pair in PAH were warrant more in‐depth exploring.

**TABLE 4:** The three miRNAs expression in h‐PASMC after PDGF‐BB stimulation

Fold change					Signal intensity
Hour (h)	0	6	12	24	Baseline
Down‐expression					
hsa‐miR‐508‐3p	1	0.82	0.37	0.21	7437
hsa‐miR‐632	1	0.86	0.51	0.33	2653
Up‐expression					
hsa‐miR‐298	1	2.17	4.06	5.97	3071

### 3.4 Down‐expression of miR‐508‐3p enhance PASMC proliferation and migration

To substantiate the role of miR‐508‐3p in PAH, we constructed PASMC/HUVEC with overexpressing or down‐regulating miR‐508‐3p by transfecting miR‐508‐3p mimics or inhibitor and analysing cell proliferation by CCK‐8 assay (Figure 5A‐B). Quantitative RT‐PCR showed that miR‐508‐3p is overexpressed in cells treated with miR‐508‐3p mimics and down‐regulated in cells transfected with inhibitor when compared with their corresponding control cells (Figure 5C‐D). The immunocytofluorescence assay results indicated that overexpression of miR‐508‐3p inhibited the proliferation of PASMC (Figure 5E‐F). Besides, we examined whether down‐regulating miR‐508‐3p would promote PASMC migration by performing wound healing assay in PASMC. The result proved that the wound healing was significantly accelerated in cells with miR‐508‐3p inhibitor when compared with the control cells (Figure 5G‐H). CCK‐8 assay exhibited that the down‐regulation of miR‐508‐3p markedly promoted proliferation of PASMC, whereas the up‐regulation of miR‐508‐3p inhibited cell growth. These data indicated that down‐regulation of miR‐508‐3p could advance the proliferation and migration of PASMC.

### 3.5 NR4A3 is the target of miR‐508‐3p in PAH

To search for the possible target gene signatures of miR‐508‐3p, the online resources miMAP (https://mirmap.ezlab.org/) and miRDB were investigated. Bioinformatic evidence suggested that one seed sequence is available between the 3′UTR of NR4A3 and miR‐508‐3p (Figure 6A). To demonstrate whether miR‐508‐3p can bind to 3′UTR of NR4A3 via the seed sequences, we constructed wild‐type and mutated seed sequences in the 3′UTR of NR4A3 mRNA. The 3′UTR sequences were cloned into the Firefly luciferase reporter plasmid. Luciferase reporter experiment displayed that when PASMC/HUVEC were cotransfected with miR‐508‐3p mimics and the wild‐type 3′UTR, the cells had significantly lower relative luciferase intensity than the cells with miRNA‐NC and the Mut‐type 3′UTR, which demonstrate that wild‐type miR‐508‐3p can bind to the seed sequences of the 3′UTR of NR4A3 mRNA to inhibit translation and intensity of luciferase (Figure 6B). To this purpose, we performed qRT‐PCR and Western blot to examine whether miR‐508‐3p inhibited the mRNA and protein expression of NR4A3. The result reveals that the cells overexpressing miR‐508‐3p show a pronounced down‐regulation of NR4A3 mRNA and protein whereas the cells less expressing miR‐508‐3p shows up‐regulated levels of NR4A3 mRNA and protein when compared with control cells in PASMC/HUVECs (Figure 6C‐D). To further corroborate our research, MCT‐PAH rat pulmonary artery tissues were used to test the mRNA and protein level of NR4A3 in vivo (n = 5), as it did align in cell experiments, the results unveil that NR4A3 represents higher expression than the control group (n = 5; Figure 6E). Next, we examined the expression levels of miR‐508‐3p in PASMC treated with the platelet‐derived growth factor‐BB (PDGF‐BB, GF149, Sigma‐Aldrich) and observed expression changes of miR‐508‐3p under different doses of PDGF‐BB in PASMC. As expected, the expression of miR‐508‐3p was down‐regulated as the dose of PDGF‐BB was increased from 10, 50 to 100 nmol/L (Figure 6F). Altogether, we confirmed that NR4A3 is a direct regulated target of miR‐508‐3p.

### 3.6 miR‐508‐3p down‐expression promotes PASMC proliferation contribute to PAH via NR4A3/MEK pathway

To further explore the biological function of the miR‐508‐3p‐NR4A3 axis, we used small‐interfering RNA to knock down NR4A3 expression. By comparing the knockdown efficiency at the protein and mRNA level, siNR4A3#2 was selected for further study (Figure 7A). Meanwhile, we observed mRNA and protein expression of NR4A3 by qRT‐PCR and Western blot when PASMC or HUVEC were treated with miR‐508‐3p inhibitor and siNR4A3#2. The result indicates that the mRNA and protein expression of NR4A3 was increased as the cells were treated with the miR‐508‐3p inhibitor, whereas they were decreased when these cells were transfected with siNR4A3#2 (Figure 7C). To unveil the mechanism underlying miR‐508‐3p promotion of PASMC proliferation, GSEA resources were performed. In light of the KEGG pathway and hallmark gene set, GSEA showed enrichment of genes chiefly involving in MAPK/ERK (MEK), mTOR and Toll‐like receptor signalling pathways (Figure 7B), and all of them was reported to act as an essential regulator in the pathogenesis of PAH; however, MAPK/ERK (MEK) signalling pathway top the list based on Normalized Enrichment Score (NES). In consequence, we interested in whether low expression of miR‐508‐3p promotes PASMC proliferation leading to PAH by activating MEK signalling, which is well established to advance the pathogenesis of PAH. Therefore, we investigated the downstream target ERK/phosphor‐ERK and Slug of the MEK pathway in PASMC or HUVEC with miR‐508‐3p overexpression or less‐expression. Our results indicate that the expression of Slug and ERK phosphorylation (p‐ERK) was increased in PASMC/HUVEC with miR‐508‐3p down‐expression and decreased in these cells with overexpression of miR‐508‐3p when compared with control cells, which is suggesting that miR‐508‐3p advances migration and proliferation via activating MEK pathway (Figure 7D). Then, the matter question is whether miR‐508‐3p mediates MEK signalling by inducing NR4A3. To further test our hypothesis that miR‐508‐3p activates MEK pathways via promoting NR4A3, we performed a rescue experiment: as merely miR‐508‐3p was down‐regulated by miR‐508‐3p inhibitor, Slug expression and p‐ERK were enhanced; when only NR4A3 was knockdown by siNR4A3#2, Slug expression and p‐ERK were decreased; when miR‐508‐3p and NR4A3 were down‐regulated sequentially, Slug expression and ERK phosphorylation were also inverted (Figure 7E). Our findings suggest that NR4A3 activates MEK pathway, regulating ERK phosphorylation and Slug expression. In summary, our results demonstrate that down‐regulated miR‐508‐3p activates MEK pathway by inducing NR4A3 to promote migration and proliferation of PASMC.

## 4 DISCUSSION

In this study, we employed two mRNA microarray profiling and two miRNA datasets involved in PAH patients from GEO platform. Based on bioinformatic analysis, we found that 185 DEGs and 172 DEMs between PAH and healthy cases and three common miRNAs. In terms of DEGs, GO and KEGG annotation were employed to formulate these molecules’ biological function and predict relevant signalling pathways. Also, the PPI network was applied to portray the complicated underlying relations of DEGs. More to the point, we identified the top 10 core genes ranked by scores and two crucial modules, which may represent the dominant factors involved in the pathogenesis of PAH. The open resources RNA predict methods enable our team to determine the responsive target genes signature of the three miRNAs, and 1266 predicted molecules were acquired. After overlapping these genes with 185 DEGs, 16 genes were generated, and we built the three miRNA‐16 mRNA regulation network. Taken a careful consideration, miR‐508‐3p‐NR4A3 pair emerging as our primary concern and was examined via experiments in vivo and vitro method.

Previous researches have reported that miR‐508‐3p show tumour‐suppressive roles in different types of cancers.^26^, ^27^, ^28^, ^29^, ^30^ As an anti‐tumour molecule, miR‐508‐3p is down‐regulated in gastric cancer (GC) and suppressed GC cell proliferation and invasion by targeting NF‐κB pathway; miR‐508‐3p expression also is reduced in ovarian cancer and targets MAPK signalling pathways. In this research, the screened miR‐508‐3p presents low expression in PAH samples. In vitro, the expression pattern of miR‐508‐3p in HPASMC or HUVEC was measured by qRT‐PCR, with the condition that these cells were treated with PDGF‐BB. As expected, miR‐508‐3p is down‐expression in both PASMC and HUVEC treated with PDGF‐BB, suggesting that miR‐508‐3p serves as a potential biomarker diagnosis and prognosis of PAH. In the present study, we demonstrated that less‐expression of miR‐508‐3p promotes HPASMC proliferation and migration by gain‐ and loss‐of‐function experiment in vitro. Notably, the CCK‐8 assay and scratch wound healing assay indicate that down‐expression of miR‐508‐3p promotes proliferation and migration of HPASMC, whereas overexpression of miR‐508‐3p shows the inverse results. More importantly, we confirmed that miR‐508‐3p promotes migration and proliferation of PASMC or HUVEC through targeting NR4A3. Initially, the ectopic expression of miR‐508‐3p decreases the mRNA and protein levels of NR4A3 in vitro; meanwhile, the down‐regulated miR‐508‐3p enhances this gene expression. Secondly, miR‐508‐3p bind to the wild‐type 3′UTR of NR4A3 as consolidated by dual‐luciferase reporter assay and bioinformatic evidence.

NR4A3 belongs to the steroid‐thyroid hormone retinoid receptor superfamily and function as critical transcriptional regulators of inflammation, differentiation, proliferation and apoptosis in cardiovascular diseases.^31^, ^32^ Previous researches have suggested that NR4A3 acts as a pro‐proliferation factor involved in atherosclerosis by enhancing cell viability and mediating cell cycle (inducing cyclin D1 and D2 expression).^32^ Some researches have reported that NR4A3 represents a tumour suppressor role in tumorigeneses such as lymphoma, breast cancer and gastric cancer by inhibiting cell viability and inducing cell apoptosis, which is hinting that NR4A3 embraces different characterize of biological functions upon specific pathogenesis of diseases.^33^, ^34^, ^35^, ^36^, ^37^ Based on the two mRNA datasets, NR4A3 is up‐regulated in PAH samples, consistent with our findings. In vivo experiment, we found that NR4A3 was up‐regulated in MCT‐PAH rat model tissues and in vitro experiment. This gene was up‐regulated in PASMC/HUVEC treated with miR‐508‐3p inhibitor, whereas NR4A3 represented down‐regulated cells subjected to miR‐508‐3p mimics compared with responsive control cells. Besides, knockdown of miR‐508‐3p promotes PASMC/HUVEC migration and miR‐508‐3p overexpression blocks these cells migration compared with responsive control cells. Therefore, we hypothesized that inhibition of miR‐508‐3p promotes migration and proliferation of PASMC/HUVEC by targeting NR4A3. Next, to unveil the underlying mechanism, we conducted GSEA analysis, which showed that enriched genes of KEGG datasets in PAH group mainly involving in MAPK/ERK pathway, of which MEK is reported to act as an essential mediator related to the progress of PAH. Therefore, we primarily focused on whether miR‐508‐3p could activate MEK signalling in PASMC/HUVEC. The outcome indicates that miR‐508‐3p can regulate both Slug expression and ERK phosphorylation of MEK signalling cascade in PASMC and HUVEC. Then, we demonstrated that NR4A3 expression activated MEK pathway, including ERK phosphorylation and Slug expression in PASMC/HUVEC. Finally, we showed that miR‐508‐3p activates MEK pathway by promoting NR4A3. These results outlined that miR‐508‐3p low‐expressed activated MEK signalling pathway via inducing NR4A3 expression.

Recent studies have shown that JAK2 phosphorylated signal transducer and activator of transcription 3 (STAT3) bound to CCNA2 promoter to boost cyclin A2 production, thereby promoting PASMC proliferation and resulting in PAH.^38^ Meanwhile, miR‐508‐3p mimics inhibited ovarian cancer cell proliferation, migration by directly targeting the 3′‐UTR of CCNA2.^26^ Therefore, whether miR‐508‐3p could mediate CCNA2 to alleviate PAH required more investigation. Synaptotagamin 5 (SYT5) is a Weibel‐Palade bodies (WPBs)‐associated Ca^2+^‐sensor, which exert its biological progress in regulating exocytosis and elevating of intracellular free Ca^2+^ concentration.^39^ The increased Ca^2+^ influx via TRPV4 contributed to the contractile, hyperproliferative and anti‐apoptotic phenotype of PASMCs.^40^ HOXA1 presents up‐regulated pattern in gastric cancer (GC) tissues. Knockdown of HOXA1 in GC cells inhibited cell proliferation, migration and invasion by inducing changes in the cell cycle with cells were increased in G1 phase, decreased in S phase and the less‐expression of cyclin D1.^41^ Mesoderm induction early response 1, family member 3 (MIER3) serves as a candidate cancer susceptibility gene. The up‐expression of MIER3 significantly inhibited human primary colorectal cancer cell (CRC) proliferation, migration partially through reduction of Sp1.^42^ ADP‐ribosylation factor‐like‐4C (ARL4C) was highly expressed in PTEN‐deficient glioblastoma (GBM) cells and tissues with its capacity to boosted the proliferation of GBM cells.^43^ To date, no relevant coverage on these molecules, such as SYT5, HOXA1, MIER3 and ARL4C, has been published to elucidate the relations between above‐talked mRNA with miR‐508‐3p, miR‐298 or miR‐632 in the pathophysiological of PAH. In consequence, the concrete regulatory mechanisms of these miRNA‐mRNA warrant further elucidated.

Endothelial cell (EC) dysfunction, such as endothelial‐to‐mesenchymal transition (EMT) and hyper‐proliferation, is widely considered as a crucial initiating factor in the pathobiology of PAH, which leads to aggravated susceptibility to apoptosis and heightened permeability.^44^ Several studies have demonstrated abnormalities in cellular bioenergetics pathways in EC. Specifically, alterations in glucose intake and utilization, together with a reduction in mitochondrial oxidative phosphorylation. Glycolysis is likely contribution to rapidly growing cells as it imparting them less dependent on oxygen consumption, thereby improving their survival abilities in a hypoxic condition and enabling EC to accommodate pro‐angiogenic stimuli by accessing to more ATP than oxidative. Pulmonary artery ECs exhibit a further tendency to lactate synthesis and aerobic glycolysis.^45^

In summary, we demonstrate that miR‐508‐3p expression is linked with diagnosis and prognosis value in PAH patients with substantiated our findings by in vivo and in vitro experiments. Furthermore, we formulate that miR‐508‐3p low‐expressed promotes PASMC migration and proliferation via targeting NR4A3 to activate MEK signalling pathway. Altogether, our study offers evidence that miR‐508‐3p is a promising biomarker for therapeutic target in patients with PAH.

## CONFLICT OF INTEREST

The authors confirm that there are no conflicts of interest.

## AUTHOR CONTRIBUTIONS

**Yi Ma:** Conceptualization (lead); Data curation (lead); Investigation (lead); Methodology (lead); Software (lead); Writing‐original draft (lead); Writing‐review & editing (lead). **Shu‐Shu Chen:** Formal analysis (supporting); Investigation (supporting); Methodology (supporting); Validation (supporting). **Fen Jiang:** Data curation (supporting); Investigation (supporting); Methodology (supporting). **Ru‐Yi Ma:** Investigation (supporting); Methodology (supporting); Software (supporting). **Huan‐Liang Wang:** Conceptualization (equal); Funding acquisition (supporting); Investigation (supporting); Methodology (lead); Project administration (equal); Writing‐original draft (supporting).

## Data Availability

The raw data that support the findings of this study are available from the corresponding author.
